# Potential genetic biomarkers predict adverse pregnancy outcome during early and mid-pregnancy in women with systemic lupus erythematosus

**DOI:** 10.3389/fendo.2022.957010

**Published:** 2022-11-16

**Authors:** Yu Deng, Yiran Zhou, Jiangcheng Shi, Junting Yang, Hong Huang, Muqiu Zhang, Shuxian Wang, Qian Ma, Yingnan Liu, Boya Li, Jie Yan, Huixia Yang

**Affiliations:** ^1^ Department of Obstetrics and Gynecology, Peking University First Hospital, Beijing, China; ^2^ Beijing Key Laboratory of Maternal Fetal Medicine of Gestational Diabetes Mellitus, Beijing, China; ^3^ Department of Medicinal Chemistry, School of Pharmaceutical Sciences, Peking University, Beijing, China; ^4^ School of Life Sciences, Tiangong University, Tianjin, China; ^5^ Department of Epidemiology and Biostatistics, School of Public Health, Peking University, Beijing, China; ^6^ Department of Rheumatology and Clinical Immunology, Peking University First Hospital, Beijing, China; ^7^ Department of Clinical Laboratory, Peking University First Hospital, Beijing, China

**Keywords:** systemic lupus erythematosus, adverse pregnancy outcome, early and mid-pregnancy, bioinformatic analysis, machine learning

## Abstract

**Background:**

Effectively predicting the risk of adverse pregnancy outcome (APO) in women with systemic lupus erythematosus (SLE) during early and mid-pregnancy is a challenge. This study was aimed to identify potential markers for early prediction of APO risk in women with SLE.

**Methods:**

The GSE108497 gene expression dataset containing 120 samples (36 patients, 84 controls) was downloaded from the Gene Expression Omnibus database. Weighted gene co-expression network analysis (WGCNA) was performed, and differentially expressed genes (DEGs) were screened to define candidate APO marker genes. Next, three individual machine learning methods, random forest, support vector machine-recursive feature elimination, and least absolute shrinkage and selection operator, were combined to identify feature genes from the APO candidate set. The predictive performance of feature genes for APO risk was assessed using area under the receiver operating characteristic curve (AUC) and calibration curves. The potential functions of these feature genes were finally analyzed by conventional gene set enrichment analysis and CIBERSORT algorithm analysis.

**Results:**

We identified 321 significantly up-regulated genes and 307 down-regulated genes between patients and controls, along with 181 potential functionally associated genes in the WGCNA analysis. By integrating these results, we revealed 70 APO candidate genes. Three feature genes, *SEZ6*, *NRAD1*, and *LPAR4*, were identified by machine learning methods. Of these, *SEZ6* (AUC = 0.753) showed the highest in-sample predictive performance for APO risk in pregnant women with SLE, followed by *NRAD1* (AUC = 0.694) and *LPAR4* (AUC = 0.654). After performing leave-one-out cross validation, corresponding AUCs for *SEZ6*, *NRAD1*, and *LPAR4* were 0.731, 0.668, and 0.626, respectively. Moreover, CIBERSORT analysis showed a positive correlation between regulatory T cell levels and *SEZ6* expression (*P* < 0.01), along with a negative correlation between M2 macrophages levels and *LPAR4* expression (*P* < 0.01).

**Conclusions:**

Our preliminary findings suggested that *SEZ6*, *NRAD1*, and *LPAR4* might represent the useful genetic biomarkers for predicting APO risk during early and mid-pregnancy in women with SLE, and enhanced our understanding of the origins of pregnancy complications in pregnant women with SLE. However, further validation was required.

## Introduction

Systemic lupus erythematosus (SLE) was a systemic autoimmune inflammatory disease that affected many organ systems (1). The incidence of SLE ranged from about 40 to 200 cases per 100,000 individuals, depending on ethnicity (2). The global prevalence of SLE was rising annually (3), and women of childbearing age were the most susceptible to SLE (4). A previous study (5) reported that pregnant women with active SLE exhibit a markedly increased risk of adverse pregnancy outcomes (APOs), such as preeclampsia (PE), abortion, preterm birth, stillbirth, renal failure and fetal growth restriction (6). Early prediction of APO risk in women with SLE was crucial for reducing maternal and infant mortality.

Previous studies (5, 7–10) had reported multiple predictors of APO in women with SLE. Mankee et al. (7) identified positive lupus anticoagulant (LAC) in the first trimester as a strong predictor of pregnancy loss. Buyon et al. (5) showed that LAC (odds ratio [OR], 8.32; CI, 3.59–19.26), antihypertensive use (OR, 7.05; CI, 3.05–16.31), a Physician’s Global Assessment score greater than 1 (OR, 4.02; CI, 1.84–8.82), and low platelet count (OR, 1.33; CI, 1.09–1.63 per decrease of 50 K cells/L) were powerful predictors of APO in pregnant women with SLE. Kim et al. (8) confirmed that soluble fms-like tyrosine kinase-1 (sFlt1) was a robust predictor of severe APO during early pregnancy in patients with SLE and/or antiphospholipid antibodies. Vicoveanu et al. (10) determined an SLE Disease Activity Index 2000 (SLEDAI-2k) score greater than or equal to 4 from the first trimester and maternal body mass index to be the top predictors for APO in women with SLE. However, even after intensive research, prediction strategies for the first and second trimester are lacking. Since the advent of sequencing technology, transcriptomics, metabolomics, and proteomics had been utilized in SLE research during pregnancy (11). These methods had greatly improved understanding of the etiology, pathogenesis, and molecular mechanisms.

This study was aimed to find potential genetic biomarkers associated with APO risk in pregnant women with SLE based on transcriptomics data. We selected the GSE108497 dataset from 8 US centers and 1 Canadian center (12) to discover APO feature genes in peripheral blood. We identified 70 APO candidate genes by performing both differentially expressed genes (DEGs) analysis and weighted gene co-expression network analysis (WGCNA). We then identified three feature genes: seizure related 6 homolog (*SEZ6*), non-coding RNA in the aldehyde dehydrogenase 1A pathway (*NRAD1*), and lysophosphatidic acid receptor 4 (*LPAR4*) by employing machine learning (ML) methods. Finally, we determined the prediction performance of *SEZ6*, *NRAD1*, and *LPAR4* using the area under the receiver operating characteristic curve (AUC) and calibration curves. ROC curve analysis and calibration curves showed that *SEZ6* exhibited satisfactory predictive performance (AUC = 0.753) and discrimination ability to predict the risk of APO. The AUC of 0.731 based on leave-one-out cross validation (LOOCV) displayed *SEZ6* had a fair predictive performance. The level of immune cell infiltration was highly linked with the progression and outcome of SLE (13, 14). We observed significant differences in the infiltration of critical immune cells, such as plasma cells, naïve CD4 T cells, and monocytes, between the SLE patients with APO (SLE-APO) and with normal pregnancy outcome (SLE-NC) groups. *SEZ6* was positively correlated with regulatory T (Treg) cells levels (*P* < 0.01), and *LPAR4* was negatively correlated with M2 macrophages abundance (*P* < 0.01). These integrative results of this study provide insight to assist in the identification of high-risk patients and enable early identification of APO in pregnant women with SLE.

## Methods

### Data sources

All gene expression profiles analyzed in this study were obtained from the GSE108497 GEO dataset, included peripheral whole blood samples from SLE-APO and SLE-NC, in 8 US centers and 1 Canadian center during September 2003 to August 2013 (12). According to the descriptions in Hong et al. (12), APO were defined as: (1) fetal deaths > 12 gestational week [GW] unexplained by chromosomal abnormalities, anatomical malformations, or congenital infections; (2) neonatal death before discharge due to preterm complications; (3) preterm delivery or termination of pregnancy < 36 GW owing to growth restriction or placental insufficiency; and (4) small for gestational age of less than the fifth percentile at birth. Peripheral blood samples collected at five specific time points (P1, < 16 GW; P2, 16–23 weeks; P3, 24–31 weeks; P4, 32–40 weeks; and P5, 8–20 weeks postpartum) were utilized for microarray analysis, as described by Hong et al. (12). Because we aimed to investigate APO in early and mid-pregnancy, we retained samples only from individuals with a GW under 24 weeks (total SLE-APO, *n* = 36; total SLE-NC, *n* = 84; P1 SLE-APO, *n* = 18; P1 SLE-NC, *n* = 45; P2 SLE-APO, *n* = 18; P2 SLE-NC, *n* = 39).

### DEGs analysis

DEGs were screened using the “limma” package in R software (v. 3.6.1) (15). The genes with an absolute log2 fold change greater than 0.5 and a *P*-value less than 0.05 were considered DEGs (16).

### WGCNA

WGCNA was applied to analyze co-expressed gene modules and identify potential genes associated with clinical traits. WGCNA was performed using the “WGCNA” package in R (17). A power of β=6 and a scale-free R^2^ = 0.9 were adopted as soft-thresholding parameters to construct a signed, scale-free co-expression gene network. Subsequently, the adjacency matrix was converted into a topological overlap matrix, and the topological overlap dissimilarity was used as hierarchical clustering input.

### Functional enrichment analysis of APO candidate genes

APO candidate genes were identified as intersecting genes of DEGs and the turquoise module in WGCNA. After obtaining the APO candidate genes, disease ontology (DO), Gene Ontology (GO), and Kyoto Encyclopedia of Genes and Genomes (KEGG) pathway analyses were performed using the “DOSE” and “clusterprofiler” packages (including”enrichGo” and “enrichKEGG” functions) in R (18, 19).

### Feature genes screening by ML

ML algorithms had advantages in multi-omics integrated analysis and provided potent data mining tools for discovering new clinical predictors (20–23). To screen feature genes, we selected random forest (RF) (24), support vector machine-recursive feature elimination (SVM-RFE) (25), and least absolute shrinkage and selection operator (LASSO) models (26). RF, SVM-RFE, and LASSO analyses were performed using the R packages “randomForest”, “caret”, and “glmnet”, respectively. RF was a non-parametric method using random decision trees for classification, and was used for many biological applications ranging from gene selection to disease prediction (24). SVM-RFE was an efficient machine learning algorithm for selection and visualization of the most relevant features through non-linear kernels (25). The LASSO regression was used for feature selection and dimension reduction (26). We identified feature genes by creating Venn diagrams of RF, SVM-RFE and LASSO results, as reported previously (27, 28).

### Evaluation of predictive performance

Receiver operating characteristic curves (ROCs) and calibration curves were used to evaluate the predictive ability of feature genes. To further examine the predictive performance of feature genes, LOOCV method was applied. Calibration curves were constructed using the “calibrate” function in “rms” package of R software with 1000 bootstrap resampling (29).

### Gene set enrichment analysis

Gene set enrichment analysis (GSEA) was performed using GSEA software (30). For each gene set of interest, an enrichment score was calculated and statistical significance was determined by comparing against the expected result of 10000 randomly permutations of the original data set (31). Gene sets with a nominal *p*-value < 0.05 and a false discovery rate (FDR) of < 0.25 were accepted as significantly enriched.

### Immune cell infiltration analysis

The CIBERSORT (32) algorithm in the “CIBERSORT” R package was used to quantify the relative proportions of infiltrating immune cells for each sample and evaluate their correlation with the expression of feature genes.

### Statistical analysis

All analyses were performed using R software (v.3.6.1). *P* < 0.05 was considered significant, *P* < 0.01 was considered highly significant, and *P* < 0.001 was considered very highly significant.

## Results

### Data preprocessing and DEG screening

**Figure 1:**
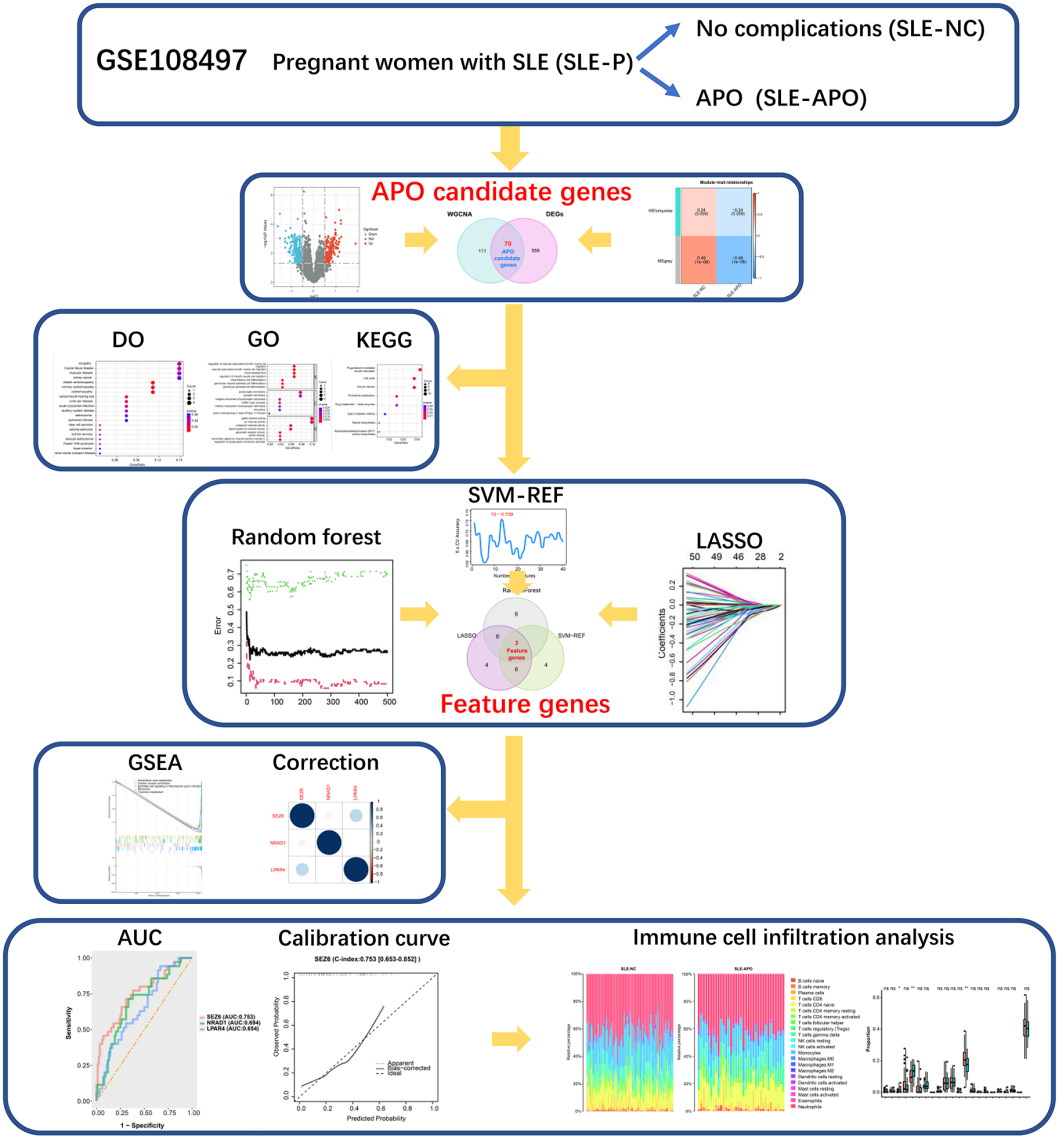
Flow chart of the data processing procedure. *P < 0.05, **P < 0.01, and ns, not significant.

**Figure 2:**
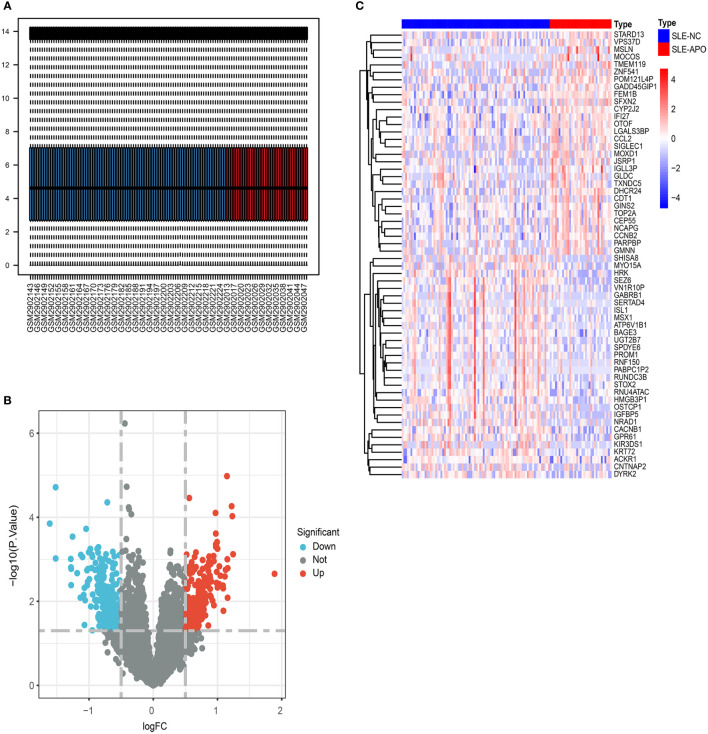
The screening of DEGs from GSE108497. **(A)** Visualization of GSE108497 after normalization. The horizontal axis represents the sample symbol and the vertical axis represents the expression values. The short black line in the box plot indicates the median value of gene expression. **(B)** A volcano plot of differentially expressed genes (DEGs). The genes with an absolute log2 fold change greater than 0.5 and *P*-value less than 0.05 were selected. Red dots represent upregulated genes while blue dots represent downregulated genes. **(C)** Heat map of the DEGs. The legend of “type” on the top right indicates the SLE patients with APO (SLE-APO) and SLE patients with normal pregnancy outcome (SLE-NC).

We analyzed data from 36 SLE-APO patients and 84 SLE-NC individuals during early to mid-pregnancy in 8 US centers and 1 Canadian center (**Figure 1**). The baseline characteristics of SLE-NC and SLE-APO groups were listed in **Supplementary Table 1**. The original expression datasets from GSE108497 showed acceptable normalization (**Figure 2A**). Based on the defined criteria, we obtained 628 DEGs between SLE-APO and SLE-NC. Of these, 321 were significantly upregulated and 307 were significantly downregulated in the SLE-APO group relative to the SLE-NC group (**Figure 2B**). Heat map construction revealed that DEGs between the SLE-APO and SLE-NC groups were distinguishable (**Figure 2C**).

### Identification of clinically significant modules and genes in WGCNA network

**Figure 3:**
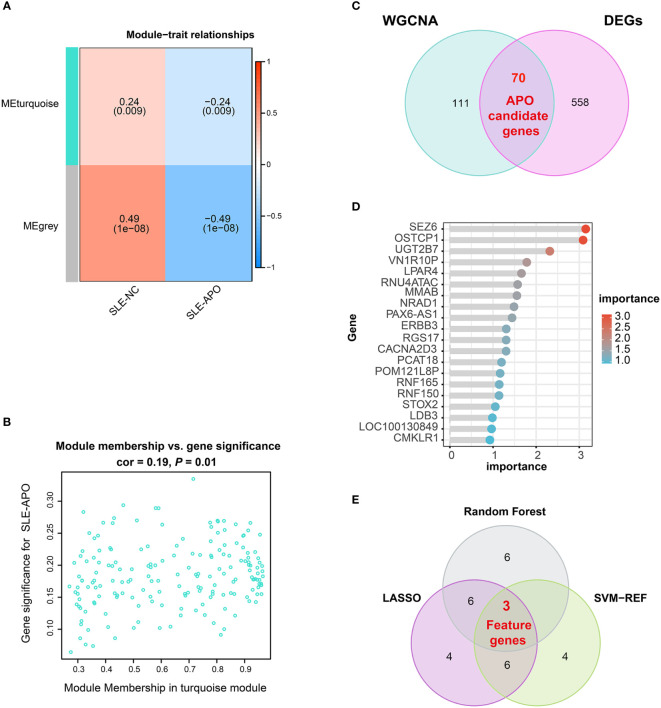
Identification of feature genes. **(A)** Heatmap of the correlation between module eigengenes and SLE-NC and SLE-APO. **(B)** Scatter plot for correlation between the gene significance for SLE-APO and Module Membership in the turquoise module. **(C)** Venn diagram illustrating the intersections of WGCNA-identified co-expressed genes and DEGs. **(D)** Genes ranked by importance for the RF algorithm. **(E)** Venn diagram displaying 3 feature intersecting genes according to the SVM-RFE, RF and LASSO analysis.

To further investigate candidate genes associated with clinical traits of SLE-APO, we performed the WGCNA analysis. To begin with, for construct a scale-free network, we set the soft threshold (β) = 6 (scale-free R^2^ = 0.90; **Supplementary Figure 1A**). Subsequently, modules with high similarity were merged and the co-expression module (turquoise) was identified (**Supplementary Figure 1B**). Correlation analysis between co-expression modules and disease revealed that the MEturquoise module was significantly negatively correlated with SLE-APO (*r* = −0.24, *P* = 0.009; **Figure 3A**). Furthermore, the correlation between the gene significance and module membership was 0.19 (*P* = 0.01; **Figure 3B**). We identified 181 genes closely associated with SLE-APO in the WGCNA network (**Figure 3C**).

### DO, GO, and KEGG enrichment analysis of APO candidate genes

To reduce the false positive rate, we performed the Venn diagram analysis and obtained 70 APO candidate genes (**Figure 3C**). To further explore the biological functions of these candidate genes, we performed DO, GO, and KEGG. DO enrichment analysis showed that APO candidate genes were significantly enriched in multiple myopathy- and cardiomyopathy- related pathways (**Supplementary Figure 2A**). Previous research found that SLE patients with APO had a higher rate of subclinical cardiovascular disease (33). GO enrichment analysis results were divided in biological process, cellular component, and molecular function (**Supplementary Figure 2B**). For biological process, 70 APO candidate genes were significantly enriched in vascular associated smooth muscle cell migration and glomerular visceral epithelial cell differentiation. For cellular component, genes were associated with the postsynaptic membrane and synaptic membrane. In the molecular function category, APO candidate genes were mainly enriched in gated channel, ion channel, potassium channel and ligand−gated ion channel activity. KEGG enrichment analysis showed that APO candidate genes were mainly enriched in progesterone**-**mediated oocyte maturation, cell cycle, and oocyte meiosis pathways (**Supplementary Figure 2C**).

### Screening and verification of feature genes *via* ML models

Three ML models (SVM-RFE, RF, and LASSO) were applied to screen feature genes from 70 APO candidate genes. The SVM-RFE model highlighted 13 genes (**Supplementary Table 2**), and the highest prediction accuracy was up to 73.9% (**Supplementary Figure 3A**). RF analysis highlighted 15 genes, with *SEZ6* ranked highest by importance score (**Figure 3D**; **Supplementary Figure 3B**, and **Supplementary Table 3**). The LASSO algorithm identified 19 genes (**Supplementary Figure 3C**, **Supplementary Table 4**). By determining the intersection of these three ML models, we identified three feature genes: *SEZ6, NRAD1*, and *LPAR4* (**Figure 3E**).

**Figure 4:**
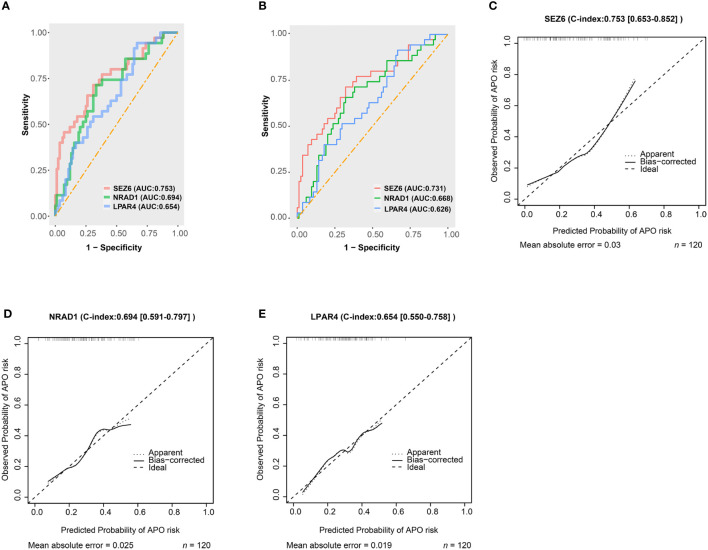
Predictive abilities for *SEZ6*, *NRAD1*, and *LPAR4*. **(A)** The ROC curve of *SEZ6*, *NRAD1*, and *LPAR4* for predicting APO risk in women with SLE during early and mid-pregnancy. **(B)** The Leave-one-out-cross-validation ROC curve of *SEZ6*, *NRAD1*, and *LPAR4* for predicting APO risk in women with SLE during early and mid-pregnancy. **(C–E)** The calibration curves for *SEZ6* **(C)***, NRAD1* **(D)** and *LPAR4* **(E)** for predicting APO risk in women with SLE during early and mid-pregnancy.

The AUC and calibration curve were the metric to evaluate the prediction performance. We evaluated the capability of the diagnostic classifier in distinguishing between SLE-APO and SLE-NC, showing that *SEZ6* had the highest in-sample predictive ability (AUC = 0.753) followed by *NRAD1* (AUC = 0.694), and *LPAR4* (AUC = 0.654) (**Figure 4A**). The LOOCV results showed that the AUC for the *SEZ6*, *NRAD1*, and *LPAR4* was 0.731, 0.668, and 0.626, respectively (**Figure 4B**). Then, the calibration curve of *SEZ6*, *NRAD1*, and *LPAR4* for predicting the risk of the SLE-APO exhibited good agreement between predictions and actual observations (**Figures 4C–4E**).

### GSEA of feature genes

All three feature genes exhibited lower expression in the SLE-APO group than in the SLE-NC group (**Supplementary Figure 4**). To investigate the biological roles of these feature genes, we performed GSEA in the low-expression group of the three feature genes.

**Figure 5:**
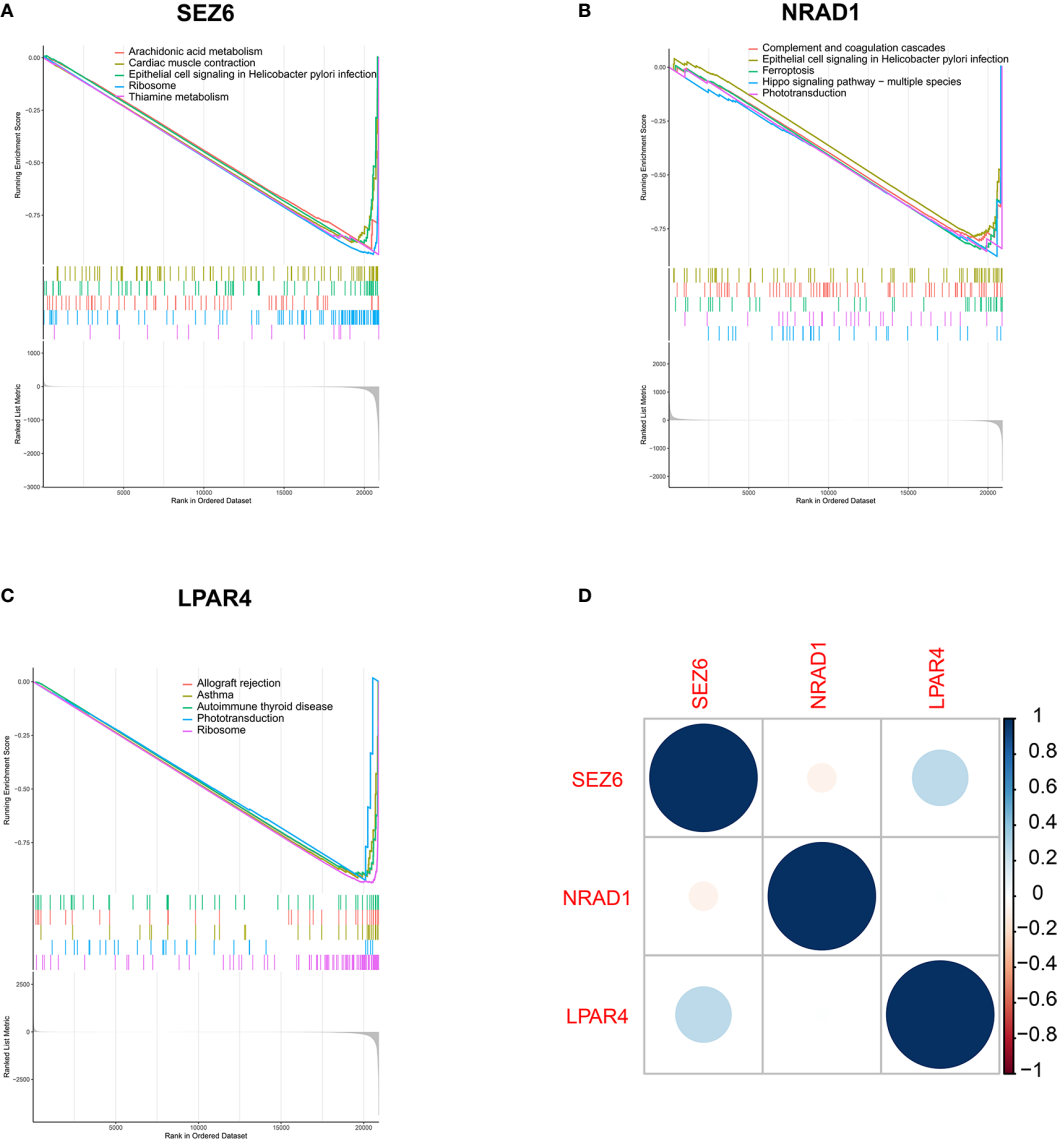
Biological pathways analysis in the low-expression group of three feature genes by GSEA analysis. **(A–C)** Down-regulated gene sets in the low-expression group of *SEZ6* **(A)**, *NRAD1* **(B)** and *LPAR4* **(C)**. **(D)** Correlation analysis of gene expression of *SEZ6*, *NRAD1*, and *LPAR4* in SLE-APO group.

The genes downregulated in the *SEZ6* low-expression group were significantly associated with arachidonic acid metabolism, cardiac muscle contraction, ribosome, thiamine metabolism and epithelial cell signaling in helicobacter pylori infection (**Figure 5A**). Genes downregulated in the *NRAD1* low-expression group were enriched in complement and coagulation cascades, ferroptosis, hippo signaling pathway–multiple species, phototransduction and epithelial cell signaling in helicobacter pylori infection (**Figure 5B**). The downregulated genes in the *LPAR4* low-expression group showed a strong correlation with allograft rejection, asthma, autoimmune thyroid disease, phototransduction, and ribosome pathways (**Figure 5C**).

Furthermore, *SEZ6* expression was positively correlated with *LPAR4* expression and negatively correlated with *NRAD1* expression in SLE-APO group (**Figure 5D**).

### Infiltrating immune cells in SLE-APO

**Figure 6:**
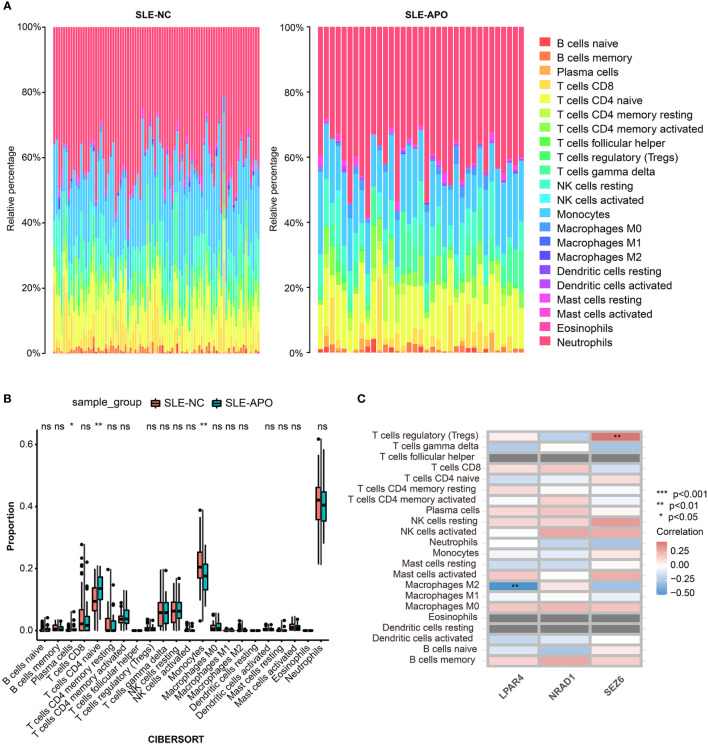
Immune cell infiltration analysis for SLE-NC and SLE-APO. **(A)** The proportion of 22 immune cells in SLE-NC and SLE-APO groups quantified by the CIBERSORT algorithm. **(B)** The difference of the proportions of 22 immune cells between SLE-NC and SLE-APO groups. **(C)** The correlation between *SEZ6*, *NRAD1*, and *LPAR4* and immune-infiltrating cells in SLE-APO group. *P < 0.05, **P < 0.01, ***P < 0.001. ns, not significant.

We performed immune cell infiltration analysis between the SLE-NC and SLE-APO groups. The fraction of immune cells in the SLE-NC and SLE-APO groups as estimated by CIBERSORT were shown in **Figure 6A**. The SLE-APO group exhibited a significantly higher proportion of plasma cells (*P* < 0.05) and naïve CD4 T cells (*P* < 0.01) and significantly lower proportion of monocytes (*P* < 0.01) than the SLE-NC group (**Figure 6B**). To further elucidate the immune-related functions of *SEZ6*, *NRAD1*, and *LPAR4*, we performed a correlation analysis between these genes and immune infiltrating cells. The results showed that *SEZ6* was positively correlated with Treg cells levels (*P* < 0.01), and *LPAR4* was negatively correlated with M2 macrophage (*P* < 0.01) abundance (**Figure 6C**).

## Discussion

SLE was a complex autoimmune disease with unpredictable severity, especially during pregnancy (34). Previous research had shown that most cases of SLE exacerbation (42.8%) occurred in the third trimester (35). Preterm premature rupture of membranes (16.6%), PE or eclampsia (15.6%), and preterm labor (12.7%) were the most common complications of SLE during pregnancy (36). Therefore, more studies were needed to identify biomarkers that could help predicting APO during early and mid-pregnancy. Recent studies had shown that two metabolites (LysoPC C22:5 and tryptophan) (37) and three proteomic predictors (SVEP1, LCAT, TGM2) (38) could accurately predict APO in pregnant woman with SLE during mid-pregnancy. Nevertheless, many challenges remained regarding the prediction of APO risk in women with SLE during the first-trimester, requiring further research.

In this study, we employed multiple gene screening modes (DEG analysis, WGCNA and ML algorithms) and identified feature genes to identify potential markers for early prediction of APO risk in women with SLE. We identified *SEZ6*, *NRAD1*, and *LPAR4* as closely related to APO in women with SLE. ROC curve analysis and calibration curves showed that *SEZ6* exhibited fair predictive performance (LOOCV-validated AUC = 0.731) and satisfactory discrimination ability to predict the risk of APO. Notably, significant differences were observed in the infiltration of critical immune cells, such as plasma cells, naïve CD4 T cells, and monocytes, between the SLE-APO and SLE-NC groups. *SEZ6* was positively correlated with Treg cells levels (*P* < 0.01), and *LPAR4* was negatively correlated with M2 macrophages levels (*P* < 0.01).

The functions of *SEZ6*, *NRAD1*, and *LPAR4* were closely linked with immune regulation or pregnancy complications. The *SEZ6* family included *SEZ6*, *SEZ6-like (SEZ6L)* and *SEZ6L2. SEZ6* was essential for nervous system development and maintenance (39). A recent study (40) showed that *SEZ6* family were novel complement regulator and that SEZ6 protein restricted C3b/inactive C3b opsonization in Chinese hamster ovary cells through classical and alternative pathways. *NRAD1*, also known as LINC00284, was localized to the nucleus and bound chromatin to affect gene expression (41). A recent study (42) demonstrated that *NRAD1* was significantly downregulated in PE placental samples. Lysophosphatidic acid (LPA) exerted a variety of biological effects, including motility and proliferation, by binding to the LPA receptor LPAR1-6. In a previous study (43), WGCNA of gene expression profiles in placental tissue from pregnant women with severe PE indicated that *LPAR4* might play key roles in PE development. mRNA expression level of *LPAR4* had been shown to be significantly higher in the placentas of PE patients than in normal placentas (44).

GSEA results revealed that most downregulated genes in the low *SEZ6*, *NRAD1*, and *LPAR4* expression groups were mainly enriched in metabolic, ribosome and immune pathways. Down-regulated genes in the *SEZ6* low-expression group were significantly enriched in arachidonic acid and thiamine metabolism. Arachidonic acid could be metabolized into prostaglandins through the cyclooxygenase pathway (45). A recent study (46) showed that prostaglandin D2 (PGD2) and PGD2 receptors (PTGDRs) induced basophil activation and infiltration in the kidney of SLE patients by mediating C-X-C theme ligand 12 (CXCL12). This study suggested that PTGDR-1 and PTGDR-2 represented promising therapeutic targets to prevent flares in SLE patients. Besides, maternal thiamine metabolism was closely related to fetal development, and thiamine diphosphate levels had been shown to be higher in preterm than in full-term infants and lower in multiples than in singletons (47). GSEA results further suggested a close relationship between *SEZ6*, *LPAR4* and ribosomal related pathway. Ribosome biogenesis and translation were particularly critical for cell growth and proliferation (48). Jie et al. (49) reported that knockdown of *ribosomal protein L39* (*RPL39*) inhibited the proliferation, migration, and invasion of trophoblast cells *in vitro*. APO was closely related to abnormal biological function of trophoblasts, since trophoblast cells were vital to placental and fetus development (50, 51). Therefore, the expression of *SEZ6* and *LPAR4* might play potential pathogenic role in APO. Furthermore, the enriched pathways of *NRAD1* and *LPAR4* included immune system pathways, such as those related to complement and coagulation cascades, asthma, and autoimmune thyroid disease. The disorder of the immune system had been associated with numerous APO (52, 53). A recent study (52) reported that activation of complement and coagulation cascades was the main pathophysiological pathway enriched in early-onset severe PE. Pregnant women with early-onset severe PE had significantly higher deposits of C5b-9 and Von Willbrand factor compared to those without complications. A recent study (53) showed that autoimmune thyroid disease was associated with increased risk of fetal growth restriction, small-for-gestation age, PE and preterm birth.

The level of immune cell infiltration was closely related to the progression and outcome of SLE (13, 14). To further investigate the role of immune cell infiltration in SLE-APO, we performed a comprehensive evaluation using CIBERSORT and conducted correlation analysis between infiltrating immune cells and *SEZ6*, *NRAD1*, and *LPAR4*. The SLE-APO group exhibited a significantly higher proportion of plasma cells (*P* < 0.05) and naïve CD4+ T cells (*P* < 0.01), and a significantly lower proportion of monocytes (*P* < 0.01) than the SLE-NC group. Previous studies (54, 55) had shown that plasma cells played a key role in the development of SLE and were positively associated with disease activity in patients with SLE. The plasma cells signature had been shown to increase in SLE pregnancies with fetal complications in the third trimester (12). Naïve CD4 T cells could differentiate into T helper cell (Th), follicular T helper and Treg subpopulations after T cell receptors activation in different cytokine environments (56). Previous study had found DNA methylation defects played an important role in the pathogenesis of SLE (57). Xin L et al. (58) found that committed Th1 CD4+ T cell differentiation blocked pregnancy induced Foxp3 expression with antigen specific fetal loss. Previous research (59) revealed significant fewer monocytes in SLE patients than in healthy individuals. Because monocytes regulated multiple immune responses, the decrease in monocytes would lead to an abnormal regulation of the immune response and played key roles in the pathogenesis of SLE (60). Monocyte activation was significant for normal pregnancy. Monocyte-derived macrophages played influential roles in trophoblast invasion, remodeling of spiral arteries, and immune homeostasis at the maternal-placental interface (61, 62).

Our results showed that *SEZ6* was positively correlated with Treg cells levels (*P* < 0.01), and *LPAR4* was negatively correlated with M2 macrophages levels (*P* < 0.01). Miscarriage, PE, and implantation failure were closely associated with the maldistribution and dysfunction of Treg cells (63). The number of decidual effector Treg cells was reduced in patients with early miscarriage (64), and clonally expanded effector Treg cells were significantly reduced in patients with PE (65), as compared to normal pregnancies. Macrophages were classically polarized into classically activated macrophages (M1) and alternatively activated macrophages (M2) in response to stimulation by different immune microenvironments (66). Studies (67, 68) had shown that a decrease in the proportion of M2-like macrophages and an increase in total macrophages was detrimental to embryo implantation. Therefore, we hypothesized that *SEZ6* and *LPAR4* might exert immunomodulatory roles in the development of APO. Further studies were needed to elucidate the complex interactions between genes and immune cells.

Our study contributes to the existing literature in three major aspects. First, because most previous studies (37, 38, 69) focused on data from the second trimester to predict the risk of APO in women with SLE, data from first trimester was urgent needed. For this reason, we specifically focused on women in early and mid-pregnancy. Second, noninvasive prenatal testing had received widespread attention since its introduction in 2011 because it only required maternal peripheral blood (70). This had led to increasing interest in developing peripheral blood-based biomarkers for pregnant women (71–73). Thus, in the present study, we performed transcriptome analysis from peripheral blood samples taken during pregnancy from nine North America centers (12). Based on the large-scale application of noninvasive prenatal testing, the validation of the findings of this study in a larger population was feasible. Third, this study employed various methods, including DEG analysis, WGCNA, and ML, to find new biomarkers. Our findings provided new ideas for future research on APO risk in women with SLE.

However, there were some limitations in our study. First, although we included a fairly large group of 120 samples from patients in nine centers, our results were exploratory in nature and require further validation. Second, while the AUC of *SEZ6* showed a satisfactory value, the results needed to be interpreted with caution considering our results were based on within-sample performance. Third, we did not evaluate the performance of our biomarkers with known APO risk factors, such as LAC status, sFlt1 values, and SLEDAI-2k scores. Fourth, the underlying mechanisms by which these feature genes affected pregnant women with SLE remained unknown. Further studies on the relationship between these genes and immune cell infiltration were also necessary.

In conclusion, we identified three predictive gene biomarkers (*SEZ6*, *NRAD1*, and *LPAR4*) of APO in pregnant women with SLE, among which *SEZ6* and *LPAR4* were closely associated with immune cell infiltration. We found *SEZ6*, *NRAD1*, *and LPAR4* might be sensitive biomarkers of APO risk in pregnant women with SLE. Our findings help to improve our understanding of the pathogenesis of APO in women with SLE, and will contribute to the development of personalized clinical management of pregnant women with SLE.

## Data availability statement

Publicly available datasets were analyzed in this study. This data can be found here: https://www.ncbi.nlm.nih.gov/geo/query/acc.cgi?acc=GSE108497.

## Author contributions

YD and HY designed the study and collected the dataset. YD, YZ, JS, JTY, and HH analyzed the dataset. YD wrote the manuscript. MZ, SW, YL, QM, BL, and JY contributed to the discussion. YD, YZ, JS, JTY, HH, and HY revised the manuscript. All authors contributed to the article and approved the submitted version.

## Funding

This work was supported by the National Key Research and Development Program of China (No. 2021YFC2700700) and the National Natural Science Foundation of China (No.81830044).

## Acknowledgments

We appreciated GEO database for providing the original study data. We thank Dr. Zifeng Cui (Department of Obstetrics and Gynecology, Peking University First Hospital) for providing amendment opinions, and Dr. Xueyan Han and Dr. Meixia Shang (Department of Medical Statistics, Peking University First Hospital) for providing statistical advices.

## Conflict of interest

The authors declare that the research was conducted in the absence of any commercial or financial relationships that could be construed as a potential conflict of interest.

## Publisher’s note

All claims expressed in this article are solely those of the authors and do not necessarily represent those of their affiliated organizations, or those of the publisher, the editors and the reviewers. Any product that may be evaluated in this article, or claim that may be made by its manufacturer, is not guaranteed or endorsed by the publisher.

## Abbreviations

APO, adverse pregnancy outcome; AUC, the area under the receiver operating characteristic curve; DEGs, differentially expressed genes; GEO, Gene Expression Omnibus; GO, gene ontology; GSEA, gene set enrichment analysis; GW, gestational week; KEGG, Kyoto Encyclopedia of Genes and Genomes; LAC, lupus anticoagulant; LASSO, least absolute shrinkage and selection operator; LOOCV, leave-one-out cross validation; *LPAR4,* lysophosphatidic acid receptor 4; ML, machine learning; *NRAD1,* non-coding RNA in the aldehyde dehydrogenase 1A pathway; OR, odds ratio; PE, preeclampsia; RF, random forest; ROC, receiver operating characteristic; *SEZ6,* seizure related 6 homolog; SLE, systemic lupus erythematosus; SLE-APO, SLE patients with adverse pregnancy outcome; SLE-NC, SLE patients with normal pregnancy outcome; SLEDAI-2k, SLE Disease Activity Index 2000; SVM-RFE, support vector machine-recursive feature elimination; Treg, regulatory T cell; WGCNA, weighted gene co-expression network analysis.
