# Supplementary material for: Potential genetic biomarkers predict adverse pregnancy outcome during early and mid-pregnancy in women with systemic lupus erythematosus
Source: Front Endocrinol (Lausanne). 2022 Nov 16;13:957010. doi: 10.3389/fendo.2022.957010 (PMC9708709; doi:10.3389/fendo.2022.957010)
Supplement: Supplementary file 1 [file DataSheet_1.pdf]

Supplementary Materials

1 Supplementary Figures

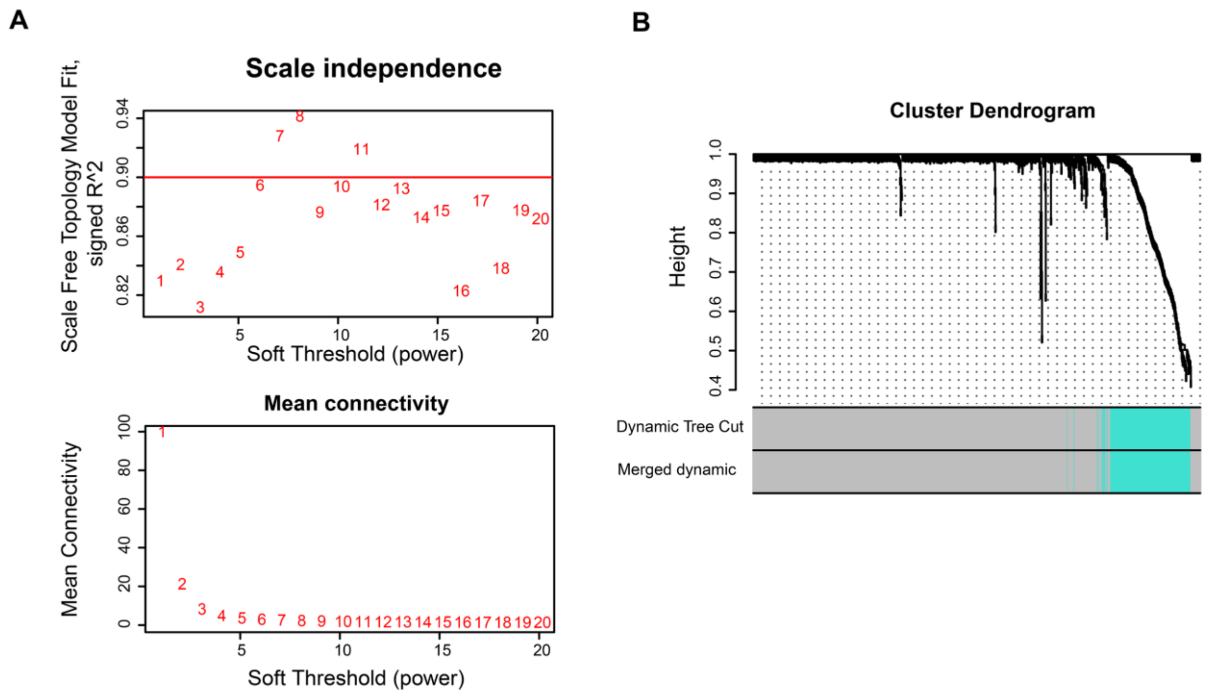

Supplementary Figure 1. Construction of the co-expression modules via WGCNA. (A) The scale-free fit index for various soft-thresholding powers (β) (top panel). The mean connectivity for various soft-thresholding powers (bottom panel). (B) Dendrogram of all differentially expressed genes clustered based on a dissimilarity measure.

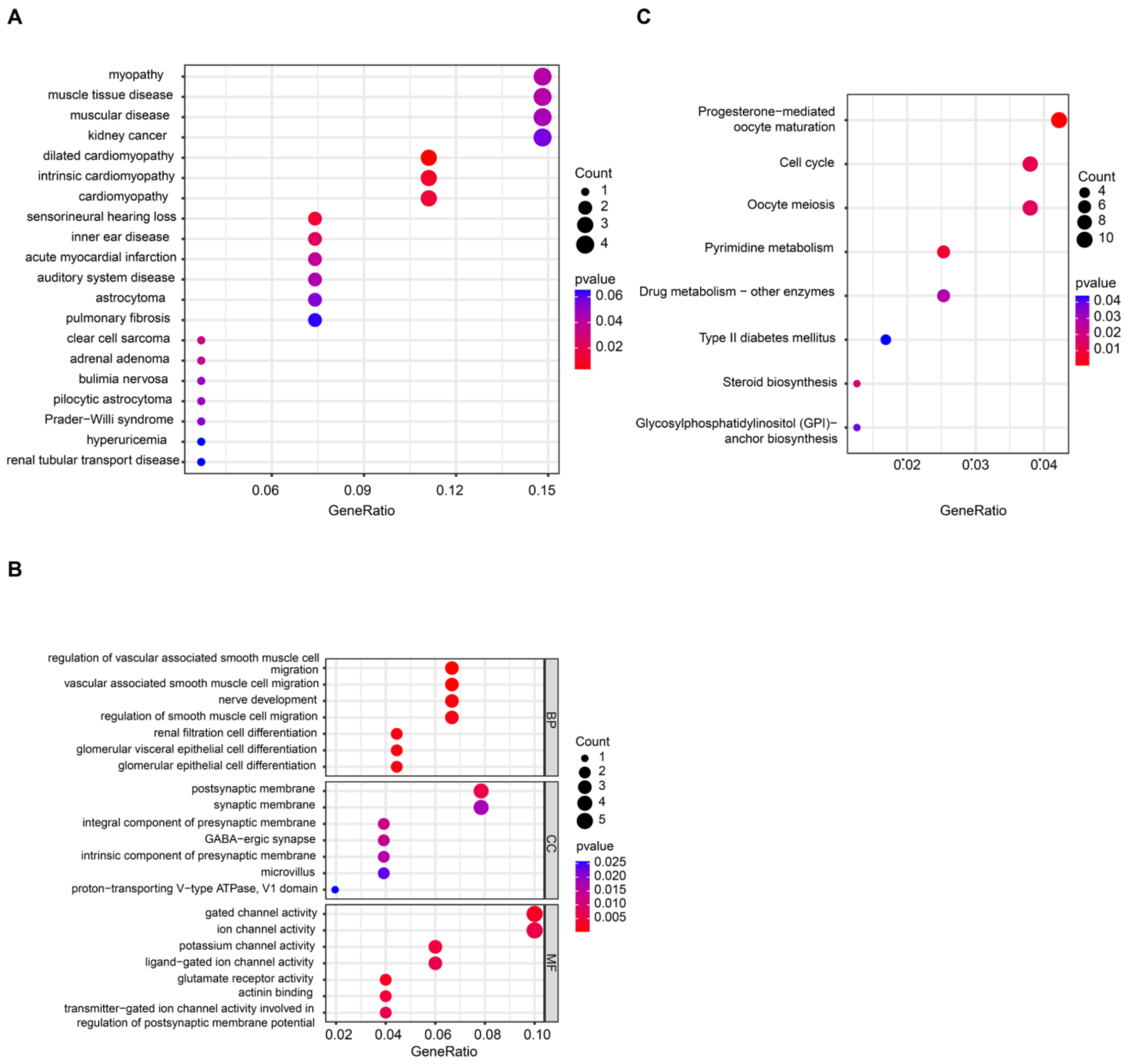

Supplementary Figure 2. The analysis of biological functions of APO candidate genes, based on DO, GO and KEGG enrichment analysis. (A) DO enrichment showed that APO candidate genes were significantly associated with myopathy and cardiomyopathy. (B) The GO enrichment results of 70 APO candidate genes. (C) The KEGG enrichment results of 70 APO candidate genes. Progesterone-mediated oocyte maturation, cell cycle and oocyte meiosis were significantly enriched in KEGG pathway enrichment analysis.

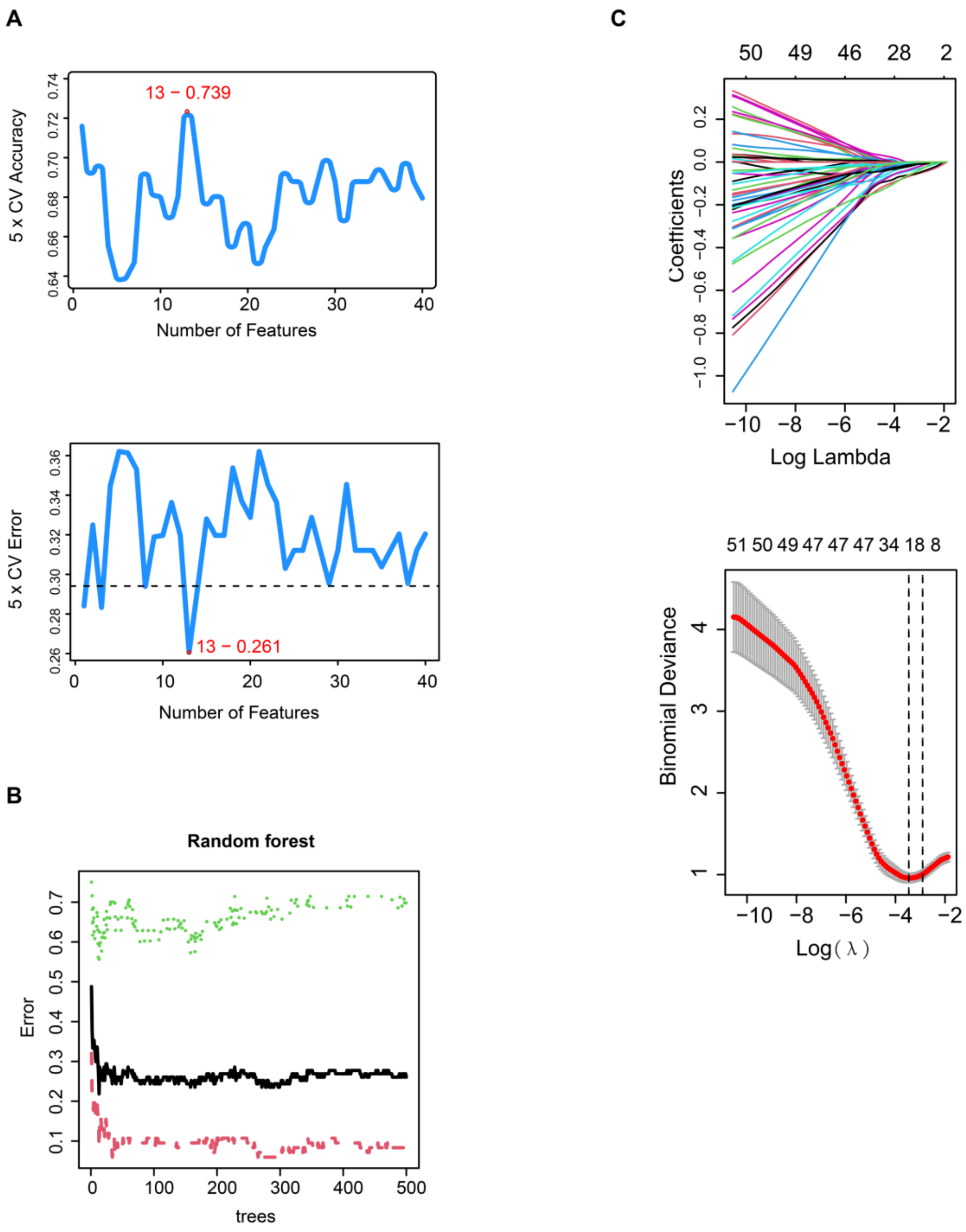

Supplementary Figure 3. Feature genes voted by three individual machine learning algorithms. (A) The screening of candidate feature genes based on SVM-RFE. The accuracy (top panel) and the error of the estimate generation (bottom panel) of the SVM-RFE algorithm. (B) The screening of candidate feature genes based on random forest. Error convergence curve according to the number of trees used in the random forest algorithm. (C) The screening of candidate feature genes based on LASSO. LASSO regression analysis for screening coefficients (top panel). The cross-validation for tuning parameter selection in the LASSO model (bottom panel).

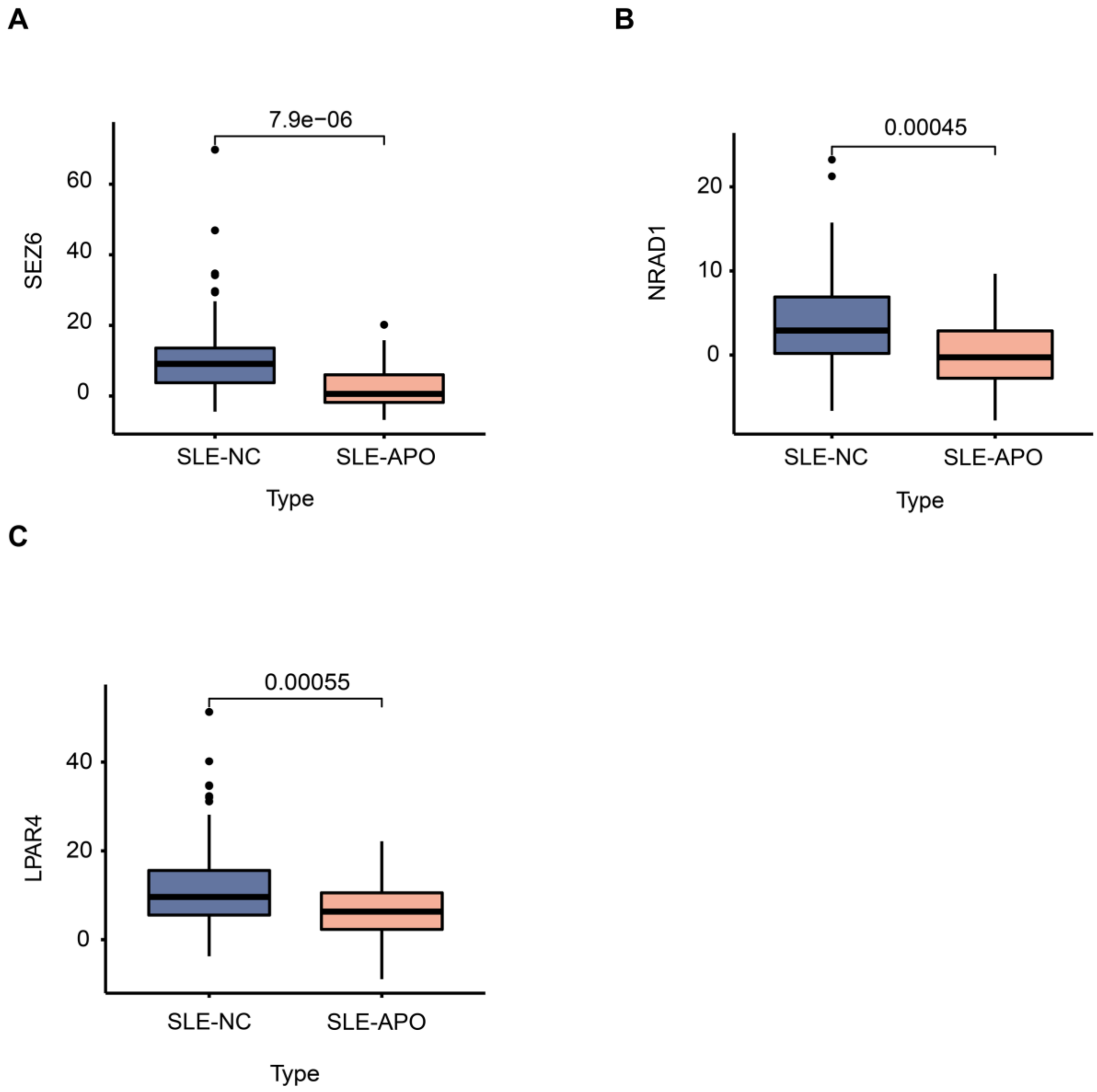

Supplementary Figure 4. The expression of three feature genes in the SLE patients with APO (SLE-APO) and SLE patients with normal pregnancy outcome (SLE-NC). *SEZ6* (A), *NRAD1* (B) and *LPAR4* (C) were low expression in SLE-APO group, compared to the SLE-NC group.

2 Supplementary Tables

Supplementary Table 1. Demographic information, laboratory values, and pregnancy outcome of SLE patients.

	SLE-APO (*n* = 36)	SLE-NC (*n* = 84)	*P*-value	Statistics
**Race**				
Caucasian, *n* (%)	17 (47.2)	58 (69.0)		
African American, *n* (%)	12 (33.3)	6 (7.1)		
Asian, *n* (%)	3 (8.3)	10 (11.9)		
Hispanic, *n* (%)	0	4 (4.7)		
Other, *n* (%)	4 (11.1)	6 (7.1)		
**Maternal age (year)**	28.1	31.0	0.005	t test
**Gestational age at collection (week)**				
P1 < 16 weeks	12.0	11.1	0.106	t test
P2:16–23 weeks	19.7	19.8	0.907	t test
**Laboratory values**				
**aPL, *n* (%)**			0.020	Fisher's exact test
No	22 (61.1)	69 (82.1)		
Yes	14 (38.9)	15 (17.9)		
**LAC, *n* (%)**			0.009	Fisher's exact test
No	26 (72.2)	77 (91.7)		
Yes	10 (27.8)	7 (8.3)		
**APOs**				
No, *n* (%)	0 (0)	84 (100)		
Yes, *n* (%)	36 (100)	0 (0)		
Fetal death, *n* (%)	13 (36.1)	0 (0)		
Neonatal Death, *n* (%)	2 (5.6)	0 (0)		
Preterm delivery<36weeks because of placental insufficiency, *n* (%)	6 (16.7)	0 (0)		
SGA<5%, *n* (%)	21 (58.3)	0 (0)		

Abbreviations: aPL, antiphospholipid antibodies; LAC, Lupus Anticoagulant.

Supplementary Table 2. The top thirteen feature genes were selected by SVM-RFE.

NO.	Gene	FeatureID	AvgRank
1	*SEZ6*	2	4.3
2	*CMKLR1*	61	8.5
3	*KDM4A-AS1*	51	10.6
4	*NRAD1*	17	11.1
5	*FLJ43315*	50	11.7
6	*LDB3*	58	11.7
7	*LSS*	56	12.9
8	*LPAR4*	34	14.2
9	*GRIK1*	40	14.4
10	*SERTAD4*	16	19.4
11	*ZNF385D*	59	20.9
12	*HOXA5*	64	21.8
13	*SLC35G6*	69	22.7

Supplementary Table 3. The top fifteen feature genes were obtained by RF.

NO.	Gene	Importance
1	*SEZ6*	3.149266202
2	*OSTCP1*	3.08787188
3	*UGT2B7*	2.316887205
4	*VN1R10P*	1.782425252
5	*LPAR4*	1.659747852
6	*RNU4ATAC*	1.570390807
7	*MMAB*	1.553915985
8	*NRAD1*	1.486305119
9	*PAX6-AS1*	1.441233043
10	*ERBB3*	1.3071092
11	*RGS17*	1.304280679
12	*CACNA2D3*	1.302633878
13	*PCAT18*	1.196853147
14	*POM121L8P*	1.165374142
15	*RNF165*	1.144758775

Supplementary Table 4. Nineteen feature genes were predicted based on LASSO.

NO.	Gene
1	*SEZ6*
2	*OSTCP1*
3	*VN1R10P*
4	*IGFBP5*

NO.	Gene
5	*RNU4ATAC*
6	*NRAD1*
7	*HMGB3P1*
8	*LPAR4*
9	*ERBB3*
10	*GRIK1*
11	*FLJ43315*
12	*PID1*
13	*LSS*
14	*CACNA2D3*
15	*LDB3*
16	*ZNF385D*
17	*PCAT18*
18	*CMKLR1*
19	*ADIPOQ*
